# Resolving Single-Cell Gene Expression by Pseudotemporal Integration of Transcriptomic and Proteomic Datasets

**DOI:** 10.1016/j.mcpro.2025.101475

**Published:** 2025-11-27

**Authors:** Craig P. Barry, Gert H. Talbo, Aiden Beauglehole, Dmitry Ovchinnikov, Trent Munro, Stephen Mahler, Kym Baker, Lars K. Nielsen, Tim R. Mercer, Esteban Marcellin

**Affiliations:** 1 Australian Institute for Bioengineering and Nanotechnology (AIBN), The University of Queensland, St Lucia, Australia; 2 The Queensland Node of Metabolomics Australia, AIBN, The University of Queensland, St Lucia, Australia; 3 Florey Institute of Neuroscience and Mental Health, University of Melbourne, Melbourne Brain Centre, Parkville, Australia; 4 Thermo Fisher Scientific, Woolloongabba, Queensland, Australia; 5 The Novo Nordisk Foundation Center for Biosustainability, Technical University of Denmark, Kgs. Lyngby, Denmark

**Keywords:** single-cell proteomics, scRNA-Seq, multiomics integration, hypoxia

## Abstract

Single-cell omics technologies, such as single-cell RNA-Seq and single-cell proteomics, offer unprecedented insights into cellular heterogeneity and dynamic regulatory processes. However, integrating these data types to construct comprehensive transcription–translation profiles remains challenging because of their distinct and complex behaviors. This study presents a novel approach using pseudotemporal cell ordering to integrate single-cell RNA-Seq and single-cell proteomics by mass spectrometry data, facilitating the analysis of transcription–translation expression dynamics. We collected longitudinal single-cell samples following hypoxia. By leveraging key markers, we constructed pseudotemporal trajectories for each data type, revealing transcriptional and translational responses to hypoxia. This profile of unified single-cell mRNA and protein expression uncovers distinct regulatory mechanisms, including an immediate transcriptomic response, followed by delayed proteomic expression. It illustrates the use of pseudotemporal integration to integrate single-cell transcriptomic and proteomic datasets to understand the cellular phenotypes under hypoxic stress and provides a framework for future investigations into transcription–translation dynamics.

## Highlights

• Hypoxia progression is defined by distinct transcriptomic and proteomic markers.
• Pseudotemporal ordering aligns hypoxia progression in scRNA-Seq and scp-MS data.
• kNN imputation enhances detection of hypoxia signals in scp-MS datasets.
• Pseudotemporal integration enables multiomic profiling of hypoxic response.

## Graphical Abstract

**Figure:**
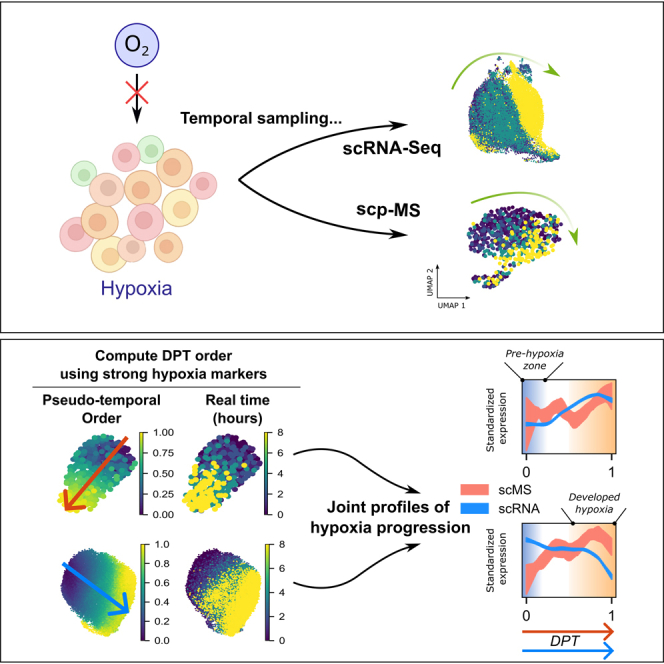

## In Brief

This study presents a method for integrating unpaired single-cell transcriptomic and proteomic data using pseudotemporal alignment. In HEK 293 cells under hypoxia, the authors show that distinct markers in each modality represent hypoxia progression. k-Nearest neighbor imputation recovers lowly represented hypoxia signals in proteomic data, enabling pseudotemporal ordering to align transcriptomic and proteomic responses. This approach supports joint analysis of gene and protein expression dynamics across the hypoxic trajectory.

The phenotype of a cell is defined by the expression of genes, which are first transcribed into mRNA and then translated into protein. This can lead to a simplistic assumption that transcript abundance directly correlates with protein abundance. However, this view ignores the temporal delay between the two and the complex network of interactions formed between proteins and the genome, which regulate gene expression through complex feedback and feedforward loops. This results in time-dependent, nonlinear relationships between transcript and protein levels. Consequently, multiple omics methods are required to understand global gene and protein expression regulation.

Measuring the transcriptome and the proteome is frequently done through bulk cell sampling (1, 2), which provides average gene and protein measurements and does not account for the heterogeneity of cell types in a sample. Single-cell approaches can determine the abundances of mRNA (single-cell RNA-Seq [scRNA-Seq]) or protein (single-cell proteomics by mass spectrometry [scp-MS]) in individual cells and afford insight into cellular heterogeneity. To increase throughput in proteomic studies, tandem mass tag (TMT)–based multiplexing strategies have been widely adopted, enabling the simultaneous analysis of dozens of cells per run. More recently, data-independent acquisition methods (3, 4) have shown rapid progress, driven by new, highly sensitive mass spectrometers that offer improved detection of low-abundance proteins and reduced missing data. These methods enable the resolution of features within populations, including rare and transient populations (5), diverging cell fates (6), and gene regulatory networks (7). This recent development of scp-MS (3, 4, 8, 9) has furthered our ability to capture the dynamics of proteomes, providing new opportunities to understand the transcription–translation dynamics in cells.

While it has been demonstrated that paired single-cell transcriptomic and proteomic measurements can be obtained from the same cell (10, 11), these approaches remain low throughput and are not yet suitable for studies aiming to identify rare or transient phenotypes. As a result, the ability to integrate unpaired single-cell gene and protein expression data remains an important challenge (12). The integration of these measurements, however, is nontrivial because of the absence of a simple linear correlation between mRNA and protein expression (13). This is further confounded by different sources of variation between scRNA-Seq and scp-MS datasets and differences in stimulus-response times in the transcriptome and proteome.

To address this challenge, we considered whether unpaired single-cell transcriptome and proteome datasets could be paired according to the expression of key gene and protein markers present in each dataset. Provided that these markers, such as cell cycle or biomarker genes and proteins, offer mutual cell-type classifications, they could be used as a shared axis to align scRNA-Seq and scp-MS datasets. Aligning these data to extract transcription–translation relationships would require a strategy that does not depend on shared features (*i.e.*, shared genes and protein markers).

Diffusion pseudotime is a tool used to construct cell trajectories by creating a pseudotemporal order across cell-type classifications in omics data (14). We therefore considered whether pseudotemporal trajectories could be used as a mutual axis for building transcription–translation profiles between mRNA and protein. This tool has been effective in identifying branched trajectories, which are often evident as diverging clusters in low-dimensional embeddings, such as uniform manifold approximation and projection (UMAP) and principal component analysis diagrams. If tightly coupled sources of variance could be isolated in unpaired scRNA-Seq and scp-MS data, they could be used for aligning pseudotemporal cell orders and transcript–translation profiles.

In this study, we collected scRNA-Seq and scp-MS data containing tightly mixed sources of biological variance, namely cell cycle and hypoxic response, to demonstrate that transcription–translation profiles can be constructed using mutual pseudotemporal axes. We used a proliferating cell nuclear antigen-fluorescent ubiquitination–based cell cycle indicator (PIP-FUCCI)–expressing (15) human embryonic kidney 293 (HEK293) cell line to identify cell cycle state and temporal sampling of hypoxia to label hypoxic response. These data demonstrated that biological sources of variance (*i.e.*, cell cycle state and hypoxic response) are defined by genes and proteins with very little overlap. Dominant markers of cell cycle and hypoxia were then used to define pseudotemporal axes for translation–transcription relationships for each source of variance. This approach allowed us to pair mRNA and protein measurements and provide integrated scRNA-Seq and scp-MS datasets. This strategy can be used to compare single-cell transcriptomic and proteomic datasets and understand regulatory mechanisms that govern cellular phenotypes.

## Experimental Procedures

### Experimental Design and Statistical Rationale

The objective of this investigation is to delineate the range of cellular responses to hypoxic stress by analyzing both the transcriptomic and proteomic landscapes at the single-cell level, using a baseline condition for reference. Initially, a culture of HEK293 cells in the exponential growth phase was sampled: once prior to the induction of hypoxia to establish a control baseline and subsequently at multiple time points during hypoxia for dynamic tracking. For the scp-MS, samples were collected at six distinct time points, with 192 cells sorted per time point into four plates, ensuring each plate represented all time points to minimize batch effects. After spectral search and quantification, cells underwent stringent filtering based on a log2-summed signal-to-noise ratio (15 < log2(ΣS/N) <17.5) and a minimum threshold of 850 proteins identified per cell to ensure data quality. In parallel, the scRNA-Seq was conducted at five corresponding time points. Cells were selected for analysis based on rigorous criteria: a minimum of 8000 counts per cell, genes detected in at least 10 cells, and a gene count ranging between 300 and 10,000. This structured experimental design, coupled with harmonized time points between scp-MS and scRNA-Seq, provided a well-labeled, high-quality dataset. This dataset facilitates an integrated analysis of transcriptional and translational responses.

### Cell Lines and Flow Cytometry

HEK293F cells that stably expressed PIP-FUCCI markers were cultured in Expi293 medium (Gibco; A1435102) at 37 °C, 130 RPM, 82% humidity, and 8% CO_2_. PIP-FUCCI fluorophores had been modified, where mMaroon1 monitored S-phase progression, whereas mAzamiGreen monitored G0/G1. Prior to culture, cells were banked in Expi293 medium and 10% dimethyl sulfoxide and stored in liquid nitrogen. Cell density and viability measurements were taken using the Vi-CELL BLU (Beckman Coulter). A fluorescence-activated cell sorting (FACS) Aria IIIu was used for cell sorting, where mMaroon1 was monitored on 633 to 660/20 (excitation–emission; “APC-A”), and mAzamiGreen was monitored on 488 to 530/30 (excitation–emission; “eGFP-A”). Settings were maintained to ensure comparative fluorescence across the plate and time points (Figs. S1 and S2).

### Bioreactor Cell Culture and Sampling

HEK–modified primary fluorescence complementation assay cells were thawed at 37 °C and resuspended in 9 ml of warmed Expi293 before centrifugation at 300 *g* for 5 min at room temperature and resuspension in 30 ml warmed Expi293 in a 125 ml Erlenmeyer flask for cultivation, as aforementioned. After reaching 4.5 × 10^6^ viable cells per ml, the culture was passaged into 120 ml Expi293 medium in a 500 ml Erlenmeyer flask and incubated as aforementioned. A bioreactor (Applikon) was inoculated with 50 ml of culture to a final volume of 550 ml and an approximate cell density of 0.4 × 10^6^ viable cells per ml and viability of >95%. For the temporal sampling of hypoxia, gas delivery to the bioreactor was suspended, and sampling time started when DO% reached 2% (Fig. S4). Bioreactor samples were taken by first purging the sampling line with a sacrificial volume of 5 ml and then taking a 5 ml sample, which was immediately cooled in an ice and water slurry. A sample was also taken prior to hypoxic insult and referred to as time 0 h. Samples were then split for scp-MS and scRNA-Seq, as described later.

### Scp-MS and scRNA-Seq Sample Preparation

Samples for scp-MS were centrifuged at 300 *g* for 2 min before resuspending in ice-cold Dulbecco's PBS (Lonza; 17-512Q). An equal number of cells from each scp-MS sample was added to a single sample and chilled on ice until cell sorting of the booster channel. The booster channel was prepared by sorting 40,200 gated cells into a PCR tube, centrifuging at 1000 *g* for 3 min, and resuspending in 20 μl of LC–MS grade water before snap freezing at −80 °C. Samples were lysed in a thermocycler by heating to 90 °C for 10 min (with a lid temperature of 105 °C) and then sonicated in a sonication bath for 15 min. Proteins were digested by the addition of 4 μl of 64 ng/μl trypsin in 640 mM triethylamine bicarbonate for approximate final concentrations of 10 ng/μl and 100 mM, respectively. Digestion was undertaken in a thermocycler for 3 h at 37 °C (lid temperature at 50 °C). Samples were cooled and centrifuged before aliquoting 3.1 μl into two 200 μl PCR tubes (SSI Bio; 323000). Peptides were then tagged by the addition of 1.6 μl of 85 mM TMTpro tags in 100% anhydrous acetonitrile (Applied Biosystems; 400060, Lot no.: VL310412) and incubation at room temperature for 1 h; one tube was tagged with 126 and the second tube with 127N. Tagging was quenched by adding 0.7 μl of 2.5% hydroxylamine and incubated for 45 min at room temperature. The 127N-tagged cells were made up to 50 cells/μl by the addition of 95 μl of 0.1% formic acid in 5% acetonitrile. From this mixture, 5 μl was transferred to the 126-tagged cells, which were then made up to 100 cells (126) and 5 cells (127N) per μl by the addition of 39.75 μl of 0.1% formic acid in 5% acetonitrile. Booster channels for each single-cell 384-well plate were prepared from the same digestion mix and combined prior to addition to plates.

Single-cell sorting for multiplexing scp-MS samples was done using the FACS Aria IIIu with a 100 μm nozzle into 384-well twin.tec PCR plates (Eppendorf) containing 1 μl of 25 fmol/μl in LC–MS grade water. PCR plates were stored at −80 °C between sorting of time-point samples. Plates were removed from the freezer, centrifuged at 1000 *g* to collect the condensate, and lysed in a thermocycler at 90 °C for 10 min and cooled to 10 °C for 30 min. Protein was digested by the addition of 0.25 μl of 50 ng/μl trypsin in 500 mM cold triethylamine bicarbonate and incubation at 40 °C for 4 h, before cooling to 10 °C for 30 min. Peptides were tagged with 1 μl of 6 mM TMT in acetonitrile and incubated for 1 h at room temperature, with subsequent quenching with 0.25 μl of 25% hydroxylamine for 30 min at room temperature. Using the Integra VOYAGER 12.5, wells were combined into multiplexed samples, and wells were flushed with 4 μl of 50% acetonitrile. The booster sample (1.2 μl) was added to each of the multiplexed samples before drying in a vacuum centrifuge and addition of 1.2 μl of 5% acetonitrile in 0.1% formic acid. Plates were kept in the fridge overnight prior to analysis. Plates were kept sealed unless reagent was being added. For scRNA-Seq, a chilled 1 ml bioreactor sample was centrifuged at 300 *g* for 2 min and subsequently resuspended in ice-cold PBS and 10% dimethyl sulfoxide before storing at −80 °C. Chromium 10X library preparation and sequencing was undertaken by the Garvan–Weizmann Centre for Cellular Genomics, using the NovaSeq 6000 S4 200 cycle kit and NextGEM SC 3′ v3.1 protocol.

### LC–MS Analysis

Single-cell samples were analyzed using Dionex UltiMate 3000 UHPLC and Orbitrap Eclipse Tribrid mass spectrometer with the FAIMS Pro, managed by Tune 3.5 and Xcalibur 4.5 (Thermo Fisher Scientific). Samples were loaded onto an Acclaim Pepmap 100 (PN 164750), which was operated at 40 °C. Peptides were resolved using a 25 cm Aurora Ultimate (AUR3-25075C18) at a flow rate of 0.2 μl/min. Flow composition was held at 4% B (80% acetonitrile and 0.1% formic acid) before ramping to 8% of 1 min. The gradient was then successively taken to 18% over 8 min, 19% over 4 min, 28% over 31 min, 38% over 20 min, 60% over 6 min, then 95% over 1 min, which was held for 23 min. Spectra were acquired using the RETICLE method developed by Furtwängler *et al*. (16). Two field asymmetric ion mobility spectrometry compensation voltages of −45 V and −60 V were used with respective cycle times of 3 s and 1.5 s. Precursors were filtered for charge states 2 to 6, and linear ion trap collision-induced dissociation MS2 spectra were searched against the GRCh38.p13 human genome (GCF_000001405.39) using real-time search, without the false discovery rate filter enabled. Upon passing the real-time search filter, Fourier transform mass spectrometer higher-energy collisional dissociation spectra were collected with a maximum injection time of 600 ms.

### Data Analysis

MS data were analyzed using Proteome Discoverer 2.5 (Thermo Fisher Scientific), where spectra were searched against the SwissProt human proteome (tax ID: 9606) with inclusion of isoforms (42,393 sequences, downloaded on November 26, 2022). The processing and consensus workflow followed the parameters established by Furtwängler *et al*. In summary, trypsin was used as the digestion enzyme, cleaving at arginine residues or lysine residues but not after proline, with a maximum of two missed cleavages allowed. Fixed modifications included TMTpro 16plex on lysine and the N terminus, whereas dynamic modifications comprised oxidation of methionine (M) and loss of methionine with acetylation. Spectra identification was performed with Sequest, using a precursor mass tolerance of 10 ppm and a fragment mass tolerance of 0.02 Da. The results were then rescored using Percolator, applying a 1% false discovery rate. No normalization or scaling was applied for reporter ion quantification in Proteome Discoverer. scp-MS and FACS (cell sorting) data were collated using the SCeptre Python package (17). For scRNA-Seq data, cellranger (18) was used for demultiplexing of dual-indexed libraries and generating feature counts using the GRCh38-2020-A reference human genome. After demultiplexing and filtering of scRNA-Seq data, total sample counts were normalized with a target sum of 10^4^ before log(x + 1) transformation and concatenation of the data. A similar approach was for scp-MS data (with exception of data concatenation), with the additional step of k-nearest neighbor (k-NN) imputation. For each single-cell proteome, a log2 sum(S/N) was calculated by summing all protein S/N measurements (as determined by Proteome Discoverer) and used for filtering in conjunction with the number of proteins identified. Cells with markedly high or low log2 sum(S/N) measurements were conservatively filtered as a potentially failed measurement or sorted doublet, respectively. Differential testing to identify markers was undertaken using a Wilcoxon test and Benjamini–Hochberg correction. Linear modeling of log-transformed scRNA-Seq and scp-MS data was undertaken using the statsmodels (0.14.0) Python library. Likelihood ratio tests were undertaken between a full model (⁓cell cycle phase + time hypoxia) and a reduced model (⁓cell cycle phase) with Holm's correction method applied. The Scanpy (1.9.1) python package was used for calculating embeddings and diffusion pseudotime.

## Results

### Imputation to Recover Hypoxia-Associated Variance in Single-Cell Proteomic Data

**Fig. 1:**
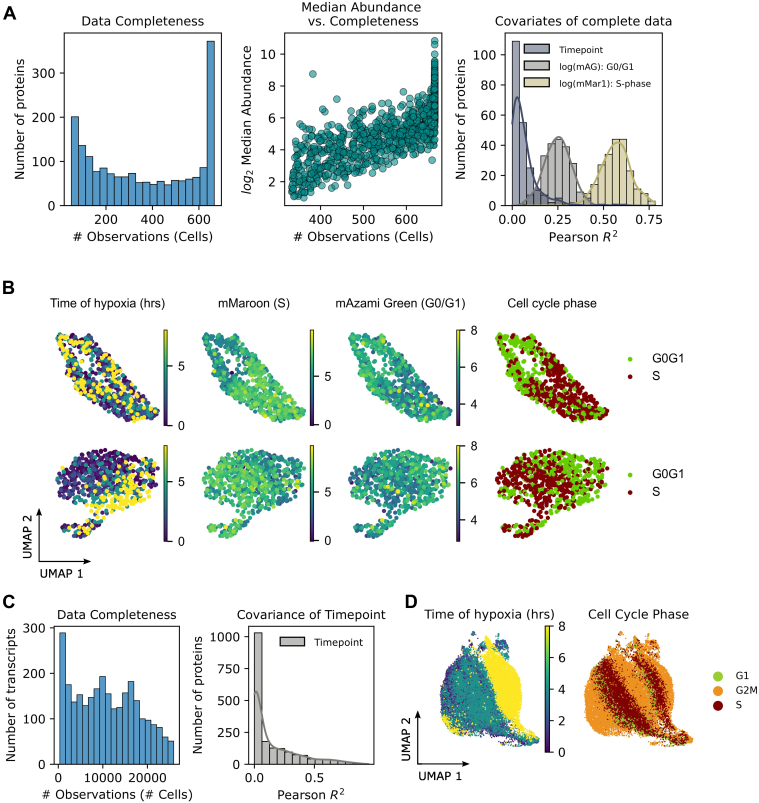
**Visual representations of scRNA and scp-MS data.***A,* histograms of scp-MS data completeness (*left*) and covariates of protein abundance (*right*). A prominent peak of 218 complete proteins was quantified in all 666 cells. Completeness *versus* abundance plots the log of summed intensities of proteins against the number of cells in which the protein is observed, demonstrating that more complete proteins are more abundant. Pearson *R*^*2*^ values are calculated between protein abundance (of the complete set) and of cell cycle marker (PIP-FUCCI) intensity and time of hypoxia. S-phase cells demonstrate the greatest covariance in the raw data, followed by G0/G1 and time point. Correlation with time is shown to be weak*. B,* UMAP plot of raw (*top row*) and imputed (*bottom row*) scp-MS data. The raw data form an elliptical shape, with temporal samples uniformly distributed throughout. PIP-FUCCI cell cycle reporters (mMaroon1, mAzamiGreen) show separation by cell cycle phase. k-Nearest neighbor imputation of scp-MS data resolves the temporal ordering of hypoxia while retaining separation in cell cycle phase. A small outgroup population appeared in scp-MS data after imputation. *C,* data completeness of scRNA-Seq data shows a distributed coverage of genes across the data, with a correlation with time of hypoxia (for all genes). *D,* UMAPs of scRNA-Seq data (without imputation) annotated by time of hypoxia and scored cell cycle phase. A small outgroup is also observed, supporting its presence in the proteome. Like the UMAP of the proteome, cell cycle phase and hypoxia groupings are distinguishable but are mixed and not spatially separated. PIP-FUCCI, proliferating cell nuclear antigen-fluorescent ubiquitination–based cell cycle indicator; scp–MS, single-cell proteomics by mass spectrometry; scRNA-Seq, single-cell RNA-Seq; UMAP, uniform manifold approximation and projection.

Cell cycle is understood to constitute a major covariate in single-cell RNA-Seq and scp-MS datasets (19). We sought to determine if biological covariates could be isolated from cell cycle variation and used to construct a mutual pseudotemporal axis for aligning scRNA-Seq and scp-MS data. To study the mixture of cell cycle and a secondary biological covariate of interest, HEK293F cells—a highly proliferative cell line—were challenged with hypoxia for temporal single-cell analyses. Briefly, cells were cultured in a bioreactor, and samples for single-cell RNA-Seq (Chromium 10X) and scp-MS were collected prior to, and during, hypoxic insult. Multiplexed scp-MS samples were prepared using the SCoPE2 workflow (8) and MS spectra were acquired using the RETICLE method (16). After cell signal filtering (Fig. S3), a median of 1016 proteins were identified per cell across 666 cells, with approximately 33% (218 total) “complete proteins” being quantified in all cells. In contrast, 23,452 genes were quantified across 4784 cells using scRNA-Seq. Endogenously expressed PIP-FUCCI markers allowed for cell cycle phase identification in scp-MS data, whereas canonical gene markers (20) were used for scoring cell cycle phase in scRNA-Seq data (21). Low-dimensional embeddings (UMAP) of these data demonstrated that cell cycle phase is a dominant structure in both scRNA-Seq and scp-MS data (Fig. 1, *B* and *D*).

An elliptical layout in the UMAP of scp-MS data showed strong alignment with PIP-FUCCI annotation, reflecting the underlying cell cycle stages (Fig. 1*B*). This was not the case for hypoxia, where a temporal trajectory was not evident in the elliptical embedding. The abundances of “complete proteins”—the most abundant proteins in the dataset (see complete *versus* abundance, Fig. 1*A*)—were analyzed for covariation with PIP-FUCCI marker intensities and time of hypoxia. This showed that the most abundant proteins in the data had the greatest covariance with cell cycle markers and little covariance with time of hypoxia (Fig. 1*A*, covariates of complete data). We also noted that data completeness distributions (Fig. 1, *A* and *C*) differ between scp-MS and scRNA-Seq data, which we attribute to the methods of measurement acquisition. While both scRNA-Seq and scp-MS methods suffer from the loss of lowly abundant transcripts and peptides, scp-MS (in this case, data-dependent acquisition) has an additional bias of acquiring spectra from more abundant proteins. These proteins are consequently more “complete proteins,” observed in all cells.

k-NN imputation of scp-MS data improved the resolution of the hypoxia-related covariance while preserving cell cycle structure, which became evident in recasted UMAP embeddings (Fig. 1*B*, *bottom row*). In contrast, scRNA-Seq data did not require imputation to visually resolve cell cycle separation from the time of hypoxia (Fig. 1*D*). Like the scp-MS data, time of hypoxia in scRNA-Seq data showed weak covariation with median transcript abundance (Fig. 1*C*). Interestingly, UMAP embeddings of scp-MS and scRNA-Seq shared a similar feature of spatially distinct large and small clusters of cells. This raised the question of whether the small cluster represented a phenotype of cell cycle or hypoxia or an alternative phenotype.

### A Subpopulation of Cells Were Not Distinctly Characterized by Cell Cycle or Hypoxia

**Fig. 2:**
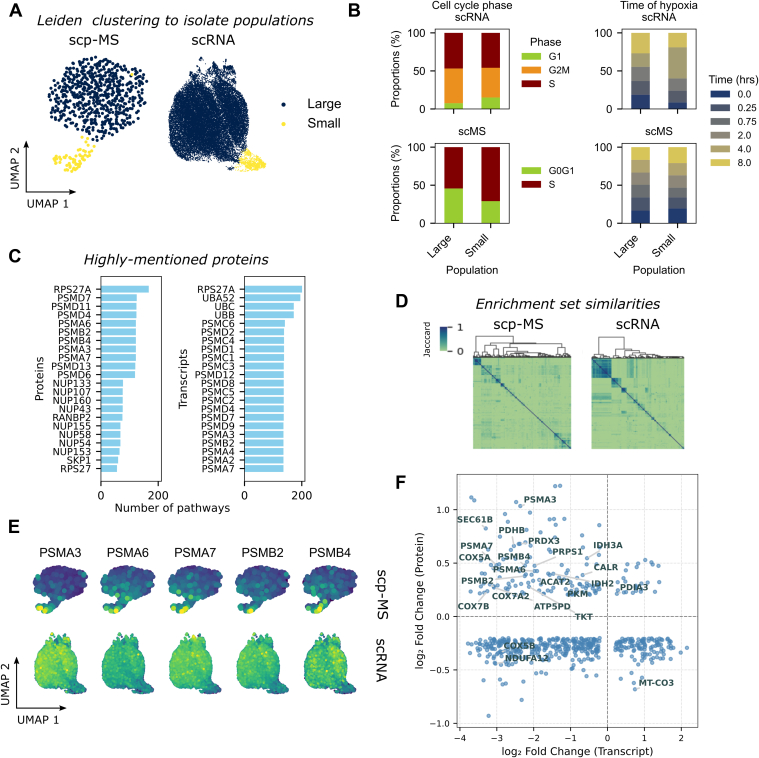
**Pathway enrichment of differentially expressed (DE) genes and proteins defines the small subpopulation of cells.***A,* UMAPs of “large” and “small” cell populations, as defined by Leiden clustering, and used for differential expression testing (Wilcoxon test). *B,* stacked boxplots showing proportions of cell cycle phases and time of hypoxia in large and small populations in scRNA-Seq and scp-MS data. There is no apparent accumulation of cycle phase or time point in the small population, suggesting this population is not defined by these covariates. *C,* the number of pathways a protein (protein) or a transcript (*right*) was mentioned in pathway enrichments of DE proteins/transcripts between the large and small populations (top 22 shown). PSMA and PSMB proteins/transcripts were highly mutual between pathways. *D,* Jaccard scores of protein and gene sets of enriched pathways cluster in blocks of high similarity, demonstrating redundant enrichments. *E,* UMAP annotated by relative expression of highly mentioned proteosome 20S components demonstrate clear differential expression in scp-MS and scRNA-Seq data, albeit in opposing directions of regulation. *F,* fold changes of mutually DE proteins and transcripts, which define the small population do not necessarily correlate in their direction of change. scp–MS, single-cell proteomics by mass spectrometry; scRNA-Seq, single-cell RNA-Seq; UMAP, uniform manifold approximation and projection.

In both scp-MS and scRNA-Seq data, a small group of cells emerged as a distinct cluster, positioned separately from a larger population of cells in a UMAP embedding (Fig. 2*A*). We note that only 62 cells were required to define this subpopulation using multiplexed scp-MS, with its presence confirmed by 928 scRNA-Seq cells. In both data, this population was comprised of all cell cycle stages and times of hypoxia, in relatively equal proportions (Fig. 2*B*). This suggests that this population is not defined by a unique cell cycle stage or stage of hypoxia. We aimed to determine if general biological features could be identified, which defined this subpopulation. Leiden clustering was used to isolate the subpopulation in scp-MS and scRNA-Seq data to identify differentially expressed (DE) transcripts and proteins (Wilcoxon–Mann–Whitney, Benjamini–Hochberg; p-adjusted ≤0.05, −0.2 ≥ log2 fold change ≥0.2). Pathway enrichment identified a substantial number of pathways, often with overlapping transcript or protein marker sets (Fig. 2, *C* and *D*). Enrichment results pointed toward significant activity in pathways related to stress responses, particularly related to high protein turnover or oxidative stress. PSMA and PSMB proteins (PSMA3, PSMA6, PSMA7, PSMB2, and PSMB4), which form the core of the 20S proteasome and are crucial for degrading misfolded proteins (22), were found to be DE in both data (Fig. 2*E*). In addition, DE proteins involved in folding and quality control (SEC61B, PDIA3, CALR, and P4HB) of newly synthesized proteins (23, 24, 25, 26) were also identified. Differences in metabolic proteins, particularly with respect to carbon metabolism (isocitrate dehydrogenase [IDH3A], PDHB, PKM, IDH2, ACAT2, PRPS1, and TKT) (27, 28, 29, 30), oxidative phosphorylation (COX5A, COX5B, COX7A2, COX7B, NDUFA9, ATP5PD, NDUFA12, and MT-CO3) (31, 32), and oxidative stress (PRDX3) (33) were also identified. We also noted that a substantial number of mutually DE proteins and transcripts showed opposite directions of upregulation and downregulation—testament to the uncorrelated nature of the transcriptome and proteome abundances (Fig. 2*F*). Taken together, increased proteosome activity could be indicative of increased protein damage because of imbalanced oxidative metabolism and its subsequent effect of oxidative stress. While it remains unclear whether the small population is a phenotype of hypoxia or cell cycle, we concluded that this distinct and aberrant phenotype should be excluded from further analysis to eliminate additional sources of variance.

### Hypoxia Impact Can Be Delineated From Cell Cycle Using Expression Markers

**Fig. 3:**
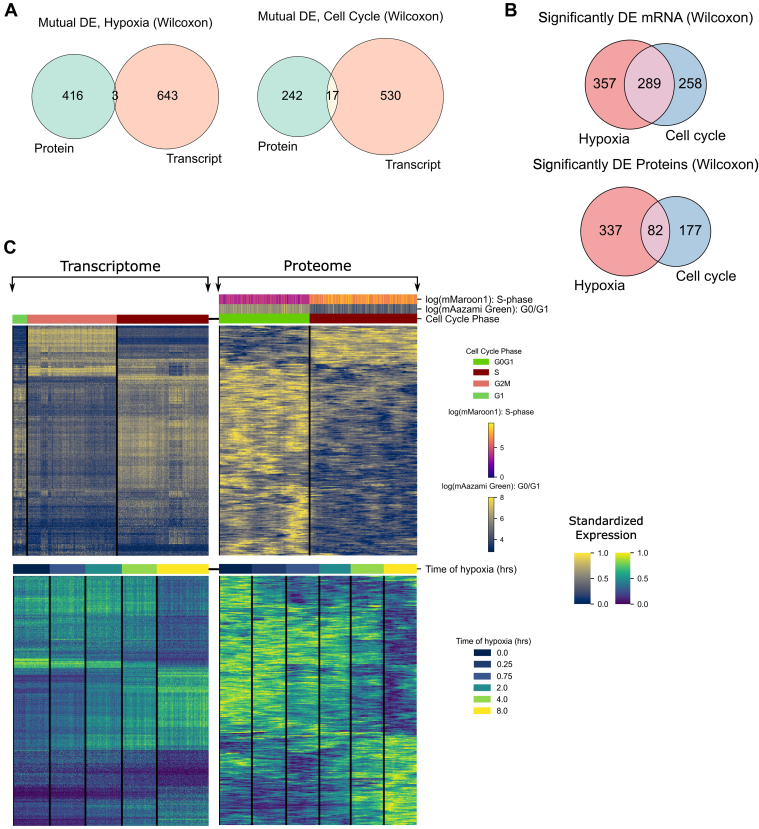
**Differentially expressed markers of cell cycle and hypoxia.***A,* Venn diagrams of protein and mRNA markers, which were identified using Wilcoxon tests between time points and cell cycle phase groups. Marker sets show little overlap, showing that mRNA and protein markers of cell cycle and hypoxia are unique. *B,* Venn diagrams of markers within each dataset show a large overlap, indicative of hypoxic-specific cell cycle responses. *C,* heatmap markers show clear upregulation and downregulation between boundaries, with various temporal regulations evident in hypoxia markers.

Further examination of the large population of cells identified sets of marker genes and proteins for cell cycle and hypoxia (Wilcoxon; Benjamini–Hochberg, p-adjusted <0.01, −0.37 ≥ log2 fold change ≥0.37). These markers were found to be largely unique between data types, irrespective of the biological covariate being assessed (Fig. 3*A*). Markers for each source of biological variation were found to be largely distinct, with only 3 (∼0.3% of all) and 17 (∼2.15% of all) transcript and protein markers being shared for hypoxia and cell cycle, respectively. While these markers were unique between data types, large overlaps were evident within data types (Fig. 3*B*). This is indicative of the large number of transcripts and proteins with a cell cycle–specific hypoxia response, which are likely responsible for the lack of distinct spatial separation in between these covariates in embeddings (Fig. 1, *B* and *D*). Grouping these markers by phase or time point revealed clear patterns of upregulation and downregulation between groupings (Fig. 3*C*).

**Fig. 4:**
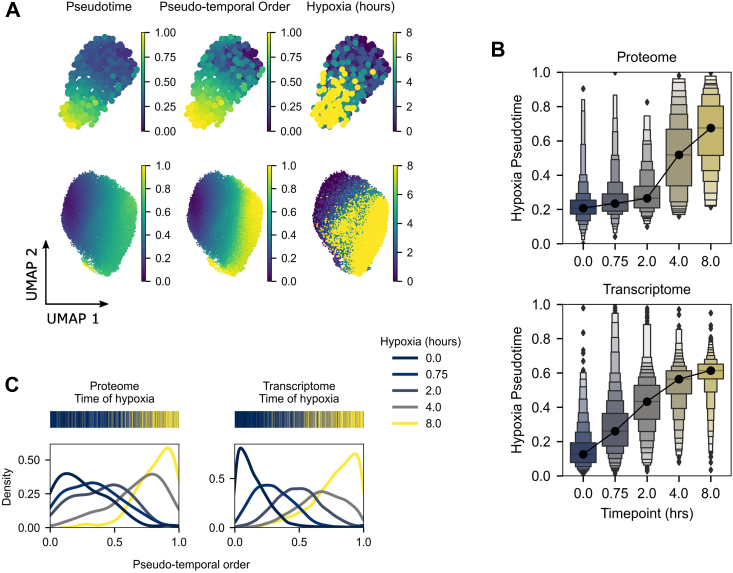
**Pseudotemporal ordering of hypoxia and embeddings of cells using markers of hypoxia.***A,* recasted UMAP plots of scp-MS (*top row*) and scRNA-Seq (*bottom row*) of pseudotime, pseudotemporal order, and real time, as calculated using the top 50 markers of hypoxia in each dataset. *B,* pseudotime with respect to real time demonstrates the lagged response of the proteome to hypoxic insult in contrast to the transcriptome. *C,* kernel density estimates of real-time (time point) distributions with respect to their pseudotemporal order, annotated by time point bands (*above*), show the heterogeneity (*i.e.*, mixing of true response times) within time points. scp–MS, single-cell proteomics by mass spectrometry; scRNA-Seq, single-cell RNA-Seq; UMAP, uniform manifold approximation and projection.

Encouraged by the ability to separate the effect of hypoxia using time point groupings, we aimed to isolate and construct a pseudotemporal trajectory of the hypoxic response in each data type. This aimed to account for the heterogenous response times of cells. The strength of transcript and protein hypoxic markers was determined using a linear modeling approach (see the *Experimental procedures* section). Briefly, complete models fitted with cell cycle phase and time-point covariates were tested against reduced models with only the cell cycle covariate (likelihood ratio test; Holm–Bonferroni method). Results were ranked by adjusted *p* value, and the top 50 hypoxia markers in each data type were used to calculate pseudotemporal cell orders. Recasted embeddings (Fig. 4*A*) revealed a clear hypoxia-dependent ordering of cells in concordance with pseudotemporal order. We anticipated that a lagged response of the proteome relative to the transcriptome could be observed in single-cell data, and we sought to use diffusion pseudotime to demonstrate this. When comparing pseudotime to real time (Fig. 4*B*), this was indeed evident; the transcriptome responded immediately (within 45 min) to hypoxic insult, whereas a global response in the proteome lagged by approximately 2 h. Examining the distributions of time points with respect to their pseudotemporal order (Fig. 4*C*) supported the expectation of heterogenous response times to hypoxia.

**Fig. 5:**
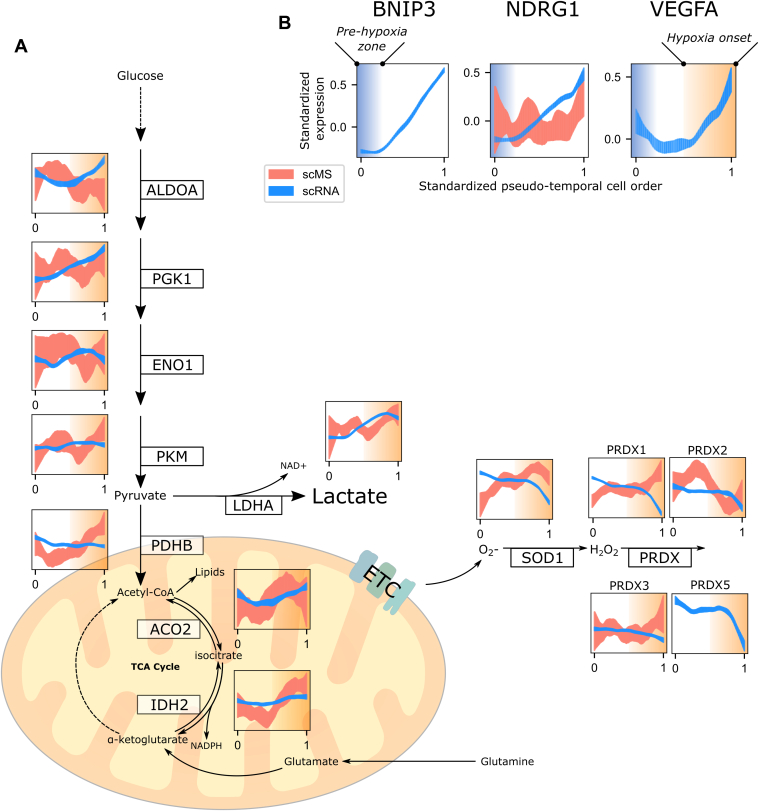
**Hallmark transcription-translation profiles of hypoxia with 95% confidence bands of standardized abundances over pseudotemporal order.***A,* canonical markers of hypoxia. Upregulation in transcription (scRNA; *blue*) of BNIP3 and NDRG1 demarcates an immediate response to hypoxia, after the prehypoxia zone, which largely contains cells that were sampled before hypoxic insult. VEGFA upregulation demarcates persistent hypoxic response, demarcated as hypoxia onset. *B,* glycolytic and antioxidant enzymes, which are upregulated after sustained hypoxia. ACO2, aconitase 2; ALDOA, aldolase, fructose-bisphosphate A; BNIP3, BCL2 interacting protein 3; ENO1, enolase 1; IDH2, isocitrate dehydrogenase 2; LDHA, lactate dehydrogenase A; NDRG1, N-myc downstream regulated 1; PDHB, pyruvate dehydrogenase E1 subunit beta; PGK1, phosphoglycerate kinase 1; PKM, pyruvate kinase M1/2; PRDX, peroxiredoxin; SOD1, superoxide dismutase 1; VEGFA, vascular endothelial growth factor A.

With the ultimate objective of using pseudotemporal trajectories to construct companion scRNA-Seq and scp-MS profiles during hypoxia, we utilized the pseudotemporal order and mutual time points between data types as a shared axis for data integration. To confirm that pseudotemporal ordering captures the dynamics of hypoxia, we examined transcription and translation dynamics, which have previously been associated with hypoxic response. Three widely reported markers of the hypoxic response, BNIP3 (34), NDRG1 (35), and VEGFA (vascular endothelial growth factor A) (36), were transcriptionally upregulated in the ordered data over the course of hypoxia pseudotime (Fig. 5*B*). We observed that transcription of BNIP3 and NDRG1 was rapidly upregulated in contrast to VEGFA. The sharp upregulation of BNIP3 and NDRG1 allowed for demarcation of the prehypoxic zone, which includes cells sampled before hypoxia, as well as cells with a delayed response to hypoxia. Similarly, we suggest that VEGFA transcriptional upregulation demarcated the zone where hypoxic response is fully established.

We investigated evidence of metabolic changes following hypoxic insult, specifically in the later zone of hypoxia onset, which are recognized as hallmarks of hypoxia. Notably, the accumulation of superoxide (O2-) during hypoxia is typically neutralized by enzymes, such as SOD1 (37) and the peroxiredoxin family proteins (PRDX1–6) (33), serving to mitigate oxidative stress. We noted that SOD1 and PRDX proteins were upregulated but not immediately upon hypoxic insult (Fig. 5*A*, *lower left*), suggesting a response to the gradual buildup of reactive oxygen species (ROS). Intriguingly, joint scRNA-Seq and scp-MS profiles indicated that the upregulation of these antioxidant proteins coincided with a decrease in their transcription. This observation implies the presence of a feedback mechanism that optimizes antioxidative activity, considering that ROS continue to serve as critical mediators of cellular signaling (38). We speculate that this occurs alongside a shift to anaerobic metabolism, where the activity of the electron transport chain diminishes, leading to less pronounced superoxide accumulation. We aimed to gain more evidence of a metabolic shift as a result of hypoxia and looked at glycolytic enzymes. This pathway is known to be upregulated during hypoxia to support ATP production to compensate for loss of oxidative phosphorylation (39, 40).

We observed increased transcription and translation of enzymes in areas of glycolysis and the tricarboxylic acid cycle, which facilitate redox (NADH and NADPH) regeneration, with upregulation occurring at different stages of hypoxia (Fig. 5*A*). Enzymes, such as ALDOA, PGK1, and ENO1, which harbor hypoxia response elements, are directly regulated by the well-studied master hypoxia regulator, hypoxia-inducible factor 1α (41). Transcriptional upregulation of these enzymes was observed relatively early upon hypoxic insult. Also characteristic of anaerobic metabolism is the production of lactate and a switch to reductive carboxylation of glutamine-derived 2-oxoglurate (42). As oxygen availability decreases, lactate dehydrogenase A (LDHA) converts pyruvate into lactate instead of channeling it into the electron transport chain. This was reflected in our data with early transcription and subsequent delayed translation (during hypoxic onset) of LDHA. Following this, to support hypoxic lipogenesis on account of redirected pyruvate, acetyl-CoA is reportedly derived from a shift to glutamine utilization, which is supported in our data by increasing IDH2 and aconitase 2 protein abundances. We also noted a dip in protein abundance in these enzymes (ALDOA, PGK1, ENO1, PKM, and LDHA) prior to the onset of hypoxia and recognize that this could be attributed to several factors, which warrant further investigation. While it is possible that these fluctuations might represent reduced protein turnover because of an abrupt shift in energy metabolism, it might also represent an area of measurement noise in the scp-MS data. Given the lag in proteome response (Fig. 4*B*) and the proximity of sample points during early hypoxia, it is also possible that protein dynamics are relatively homogenous in this region, resulting in less distinct pseudotemporal cell orders. We speculate that extending measurements beyond the 8-h duration of this study might further clarify the later stages of enzyme upregulation and diminish the apparent initial fluctuations in protein profiles. In addition, as k-NN smoothing was required to recover hypoxia-associated variance in our data, we tested whether the inferred hypoxia axis remained consistent when using unimputed data. Profiles corresponding to more abundant proteins showed strong similarity between imputed and unimputed datasets (Fig. S7), supporting that the main hypoxia trajectory is robust to imputation.

Taking this evidence together, our approach of isolating strong markers of hypoxia from the tightly coupled covariate of cell cycle to create a mutual axis of pseudotemporally ordered single cells was validated by the dynamics observed in glycolysis and ROS detoxification pathways. Moreover, this revealed possible feedback transcription–translation of SOD1- and PRDX-family proteins, which, to our knowledge, have not been characterized.

## Discussion

In this study, we generated single-cell scRNA-Seq and scp-MS data containing tightly mixed sources of biological variance, namely cell cycle and hypoxic response. The data revealed that biological sources of variance (*i.e.*, cell cycle state and hypoxic response) are defined by distinct sets of genes and proteins with minimal overlap. These dominant markers of cell cycle and hypoxia were then used to establish pseudotemporal axes for translation–transcription relationships for each source of variance.

Single-cell methods allow for the identification of cell types within heterogenous samples. These arise from several mixed sources of biological variance, such as the cell cycle, metabolic adjustments, and environmental interactions. Given the intrinsic differences between scRNA-Seq and scp-MS measurements, we anticipate that these measurements might not uniformly represent the same biological effects. For instance, one biological effect, such as the cell cycle, might predominate in one type of data (*e.g.*, scp-MS), whereas another effect, such as metabolic adjustment, might be more influential in another type of data (*e.g.*, scRNA-Seq). To examine this, we collected time-course scRNA-Seq and scp-MS data of HEK293 cells, which had been subjected to hypoxia. These cells expressed PIP-FUCCI markers, which afforded identification of cell cycle state in scp-MS data, whereas cell cycle was annotated in scRNA using canonical gene expression markers.

Dimensionality reduction of scp-MS data initially showed that cell cycle was a highly dominant and sole feature of the proteome, before k-NN imputation recovered the variance associated with temporal hypoxia. This implied that the variance associated with hypoxia in scp-MS data was contained in sparsely quantified proteins and was overshadowed by the more abundant proteins, which correlated with cell cycle trajectory. It is likely that this overshadowing occurs because many proteins increase in abundance as cell volume increases, correlating with cell cycle progression. We also found this to be true in scRNA-Seq data, where few mRNAs showed strong covariance with hypoxia duration. However, we noted that hypoxia duration and a small cluster of cells—only revealed in scp-MS data postimputation—were visually distinguishable in scRNA-Seq UMAP embeddings without k-NN imputation.

This may be a consequence of the number of cells analyzed in each dataset. The larger number of cells analyzed in the scRNA-Seq dataset might facilitate a more definitive characterization of the sparse gene set defining hypoxia, thereby establishing a clearer hypoxia-related axis. In addition, the data acquisition method for scp-MS is likely to play a significant role. We used data-dependent acquisition, which prioritizes the sequencing of the most abundant spectra in the sample. This choice partially supports our hypothesis that fundamental differences in measurement techniques result in dominant effects in one data type (*i.e.*, cell cycle in scp-MS data) compared with the other. Moreover, this highlights a current limitation of scp-MS: the throughput of scRNA-Seq methods is significantly higher, yielding a greater number of single-cell measurements. This does not imply that one data type can replace the other, as we demonstrated that the same biological phenomena can be characterized by distinct protein and mRNA marker sets.

Protein and mRNA markers of cell cycle and hypoxia response were identified through statistical hypothesis testing between data labels. As noted earlier, both cell cycle and hypoxia were characterized by distinct sets of proteins and mRNAs, with minimal overlap between them. This highlights the challenge of integrating single-cell multiomics data: not only are transcription and translation profiles inherently different, but the lack of correlated gene–protein dynamics further complicates the direct integration of these datasets.

We sought to overcome this by utilizing these unique marker sets to construct independent trajectories of hypoxia in the form of a pseudotemporal cell order. These were then used as a mutual axis between datasets. This approach accounts for heterogeneity in temporal responses and reveals a range of transcription–translation relationships. The trajectories we established using this approach were supported by the expected regulation in canonical markers of hypoxia as well as glycolytic and antioxidant enzymes. However, we observed that transcription dynamics were better resolved than translation dynamics. This discrepancy is likely because of the aforementioned limitation of scp-MS data, where fewer single-cell observations result in broader protein abundance estimates.

Single-cell omics datasets promise the opportunity to understand the expression of genes that define cellular phenotypes. However, the expression of genes is dependent on both transcriptional and translational processes that are dynamically regulated, and the integration of gene and proteomics datasets at single-cell resolution remains challenging. In this study, we developed a strategy that uses pseudotemporal cell orders to serve as a mutual axis to integrate scRNA-Seq and scp-MS datasets and enable integrated analysis of transcriptome and proteome datasets at a single-cell resolution.

While our approach demonstrates a practical framework for integrating scRNA-Seq and scp-MS data, several limitations remain. The effects of batch correction and imputation methods, along with the choices of their parameters, have not yet been thoroughly evaluated. A deeper assessment is needed to understand how these preprocessing steps may influence the consistency and robustness of the integration results. In addition, this approach was developed for datasets that reflect a progressive transition from one state to another, where biologically meaningful markers can be identified to construct a continuous trajectory. It is neither designed for datasets composed of discrete, nonoverlapping clusters nor has it been tested in settings involving branching trajectories or cell fate bifurcations. Future work will be required to adapt or extend the method for use in more complex cellular systems with nonlinear or multilineage progression.

Despite these limitations, our current analysis highlights the strengths of the method when applied to a well-defined biological system with continuous transitions. Using a multimodal dataset comprising scRNA-Seq and scp-MS measurements of HEK293F cells, we have shown that unique features of the transcriptome and proteome define sources of biological variation. This was anticipated as a feature of biological regulation, where transcription and translation operate under different mechanisms of regulation. Our analysis successfully isolated specific sources of variation, such as the hypoxic response, to construct pseudotemporal trajectories that illustrate the progression of the variation in question. This approach was validated by the expected dynamics of canonical hypoxia markers alongside antioxidant and glycolytic enzymes, confirming their roles in cellular adaptation to hypoxia. More importantly, our findings suggest that this integrative approach could be further employed to explore—and potentially reveal novel—insights into transcription–translation interactions and their implications for phenotype regulation. Such investigations could lead to a deeper understanding of the complex orchestration of gene expression and protein synthesis that underpins cellular function and diversity.

## Conclusion

Single-cell omics datasets promise the opportunity to understand the expression of genes that define heterogenous phenotypes. However, the expression of genes is dependent on both transcriptional and translational processes that are dynamically regulated, and the integration of mRNA and proteomics datasets at single-cell resolution remains challenging. In this study, we report a strategy that uses pseudotemporal cell orders to serve as a mutual axis to integrate scRNA-Seq and scp-MS datasets and enable an integrated analysis of transcriptome and proteome datasets at a single-cell resolution.

Using unpaired scRNA-Seq and scp-MS datasets of HEK293F cells undergoing hypoxia, we have demonstrated that sources of biological variance in the transcriptome and proteome can be defined by unique biological markers. This was anticipated as a feature of biological regulation, where transcription and translation operate under different mechanisms of regulation. Our analysis successfully isolated specific sources of variation, such as the hypoxic response, to construct pseudotemporal trajectories that illustrate the progression of the variation in question. This approach was validated by the expected dynamics of canonical hypoxia markers alongside antioxidant and glycolytic enzymes, confirming their roles in cellular adaptation to hypoxia. More importantly, our findings suggest that this integrative approach could be further employed to explore—and potentially reveal novel—insights into transcription–translation interactions and their implications for phenotype regulation. Such investigations could lead to a deeper understanding of the complex orchestration of gene expression and protein synthesis that underpins cellular function and diversity.

## Data Availability

MS raw data and Proteome Discoverer search files and results have been deposited to ProteomeXchange under the identifier PXD053053. Raw scRNA-Seq data are accessible from a Gene Expression Omnibus repository using the accession number GSE273172. FACS files of cells sorted for scp-MS, and processed scp-MS and scRNA-Seq data, can be accessed from a Zenodo repository: https://zenodo.org/records/12615623. Complete data processing and figure generation workbooks can be found at github.com/AIBN-SysBio/sc-Hypoxia.

## Supplemental Data

This article contains supplemental data.

## Conflict of Interest

K. B. is employed by Patheon by ThermoFisher Scientific. All other authors declare no competing interests.
