# Supplementary material for: Resolving Single-Cell Gene Expression by Pseudotemporal Integration of Transcriptomic and Proteomic Datasets
Source: Mol Cell Proteomics. 2025 Nov 27;25(1):101475. doi: 10.1016/j.mcpro.2025.101475 (PMC12892064; doi:10.1016/j.mcpro.2025.101475)
Supplement: Supplementary figures [file mmc2.docx]

**Additional file 1: Supplementary figures**

**Resolving single-cell gene expression by pseudo-temporal integration of transcriptomic and proteomic datasets**

Craig P. Barry^1^, Gert H. Talbo ^1,2^, Aiden Beauglehole^1^, Dmitry Ovchinnikov^3^, Trent Munro^1^, Stephen Mahler^1^, Kym Baker^5^, Lars K. Nielsen^1,4^, Tim R. Mercer^1^, Esteban Marcellin^1,*^

1. Australian Institute for Bioengineering and Nanotechnology (AIBN), The University of Queensland, 4072 St. Lucia, Australia.
2. The Queensland Node of Metabolomics Australia, AIBN, The University of Queensland, 4072 St. Lucia, Australia.
3. Florey Institute of Neuroscience and Mental Health, University of Melbourne, Melbourne Brain Centre, Parkville, Australia
4. The Novo Nordisk Foundation Center for Biosustainability, Technical University of Denmark, DK-2800 Kgs. Lyngby, Denmark.
5. Thermo Fisher Scientific, Woolloongabba, Queensland, Australia.


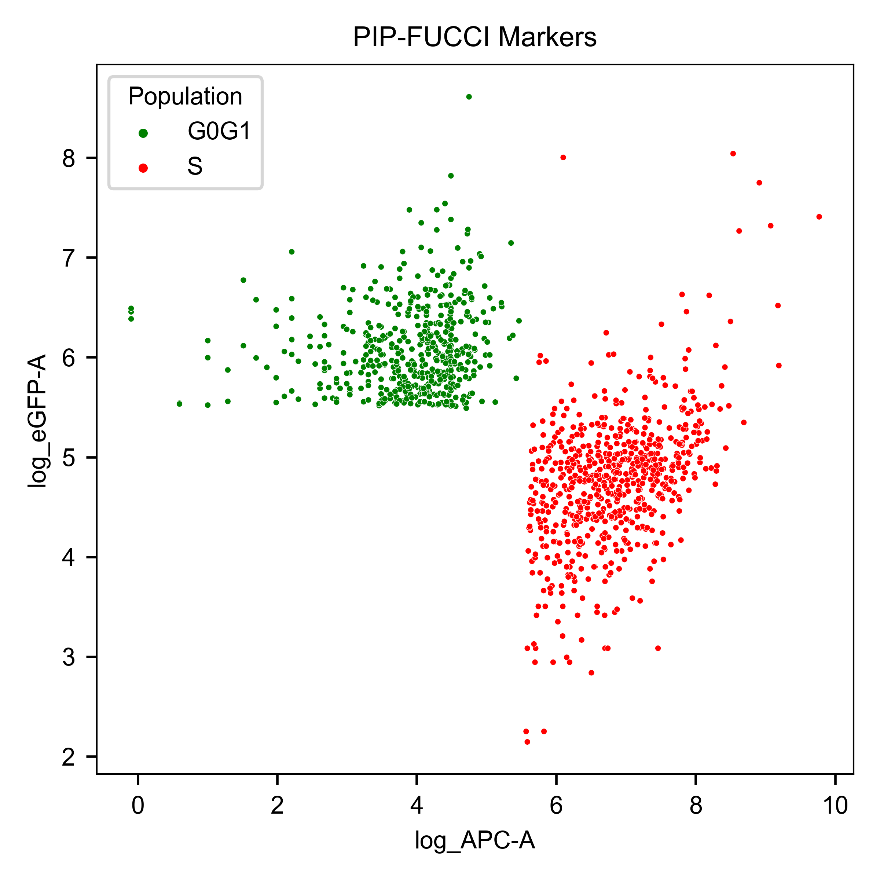


**Fig. S1. Sorted cells and PIP FUCCI marker fluorescence intensities for scp-MS.**

‘eGFP-A’ and ‘APC-A’ channels were used to measure mAzami green and mMaroon (PIP-FUCCI) markers of cell cycle stage, respectively.


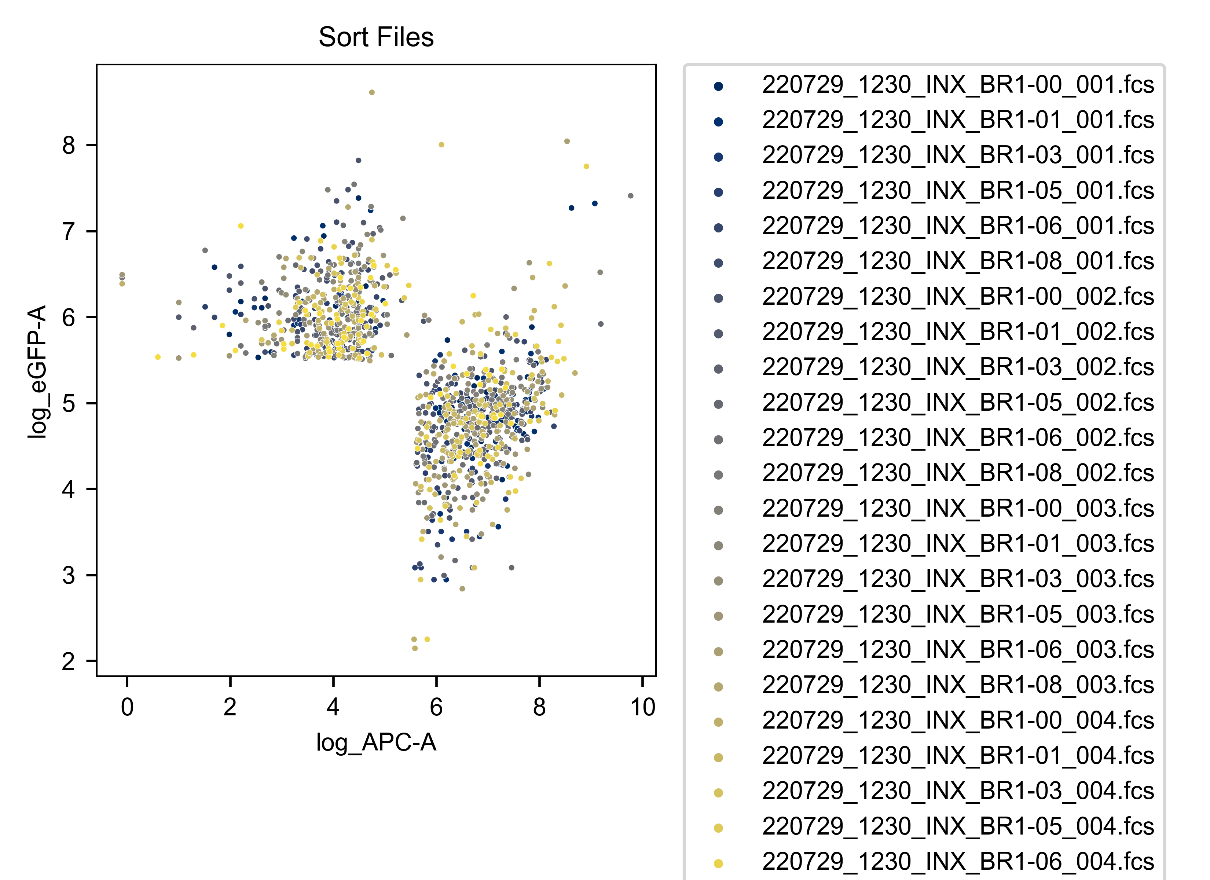


**Fig. S2. Sorted cells for scp-MS colored by 384-well plate (001-004) and sample timepoint number (00, 01, 03, and 08).**

Sorted cells show homogenous – well mixed – cell cycle stage across plates and hypoxia timepoints.


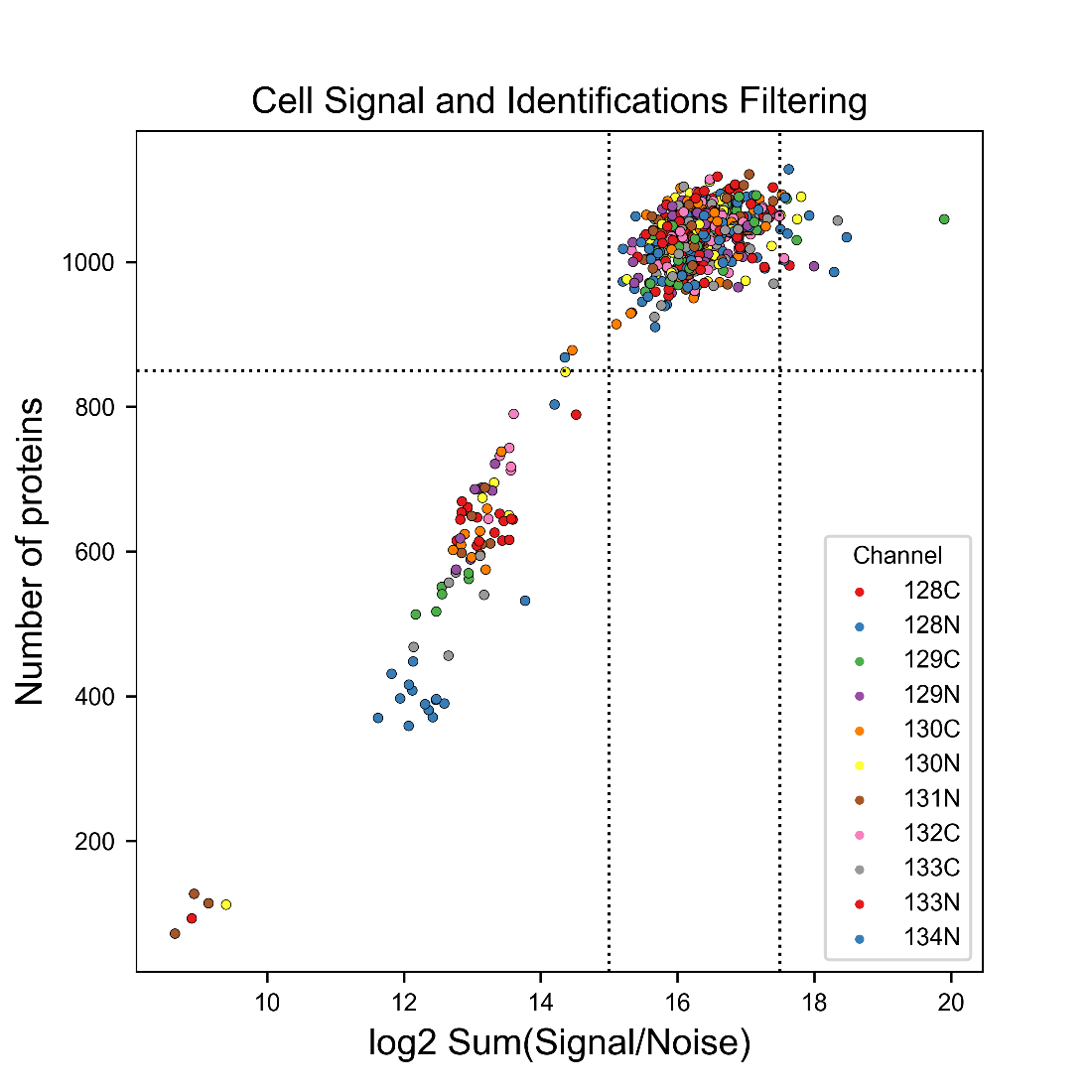


**Fig. S3. Cell filter for single cell proteomics data.**

Cells analyzed by scp-MS were filter by the number of proteins and the logged sum of total signal-to-noise for each cell. Cells with high signal-to-noise were taken as ‘doublet’ and filtered out.


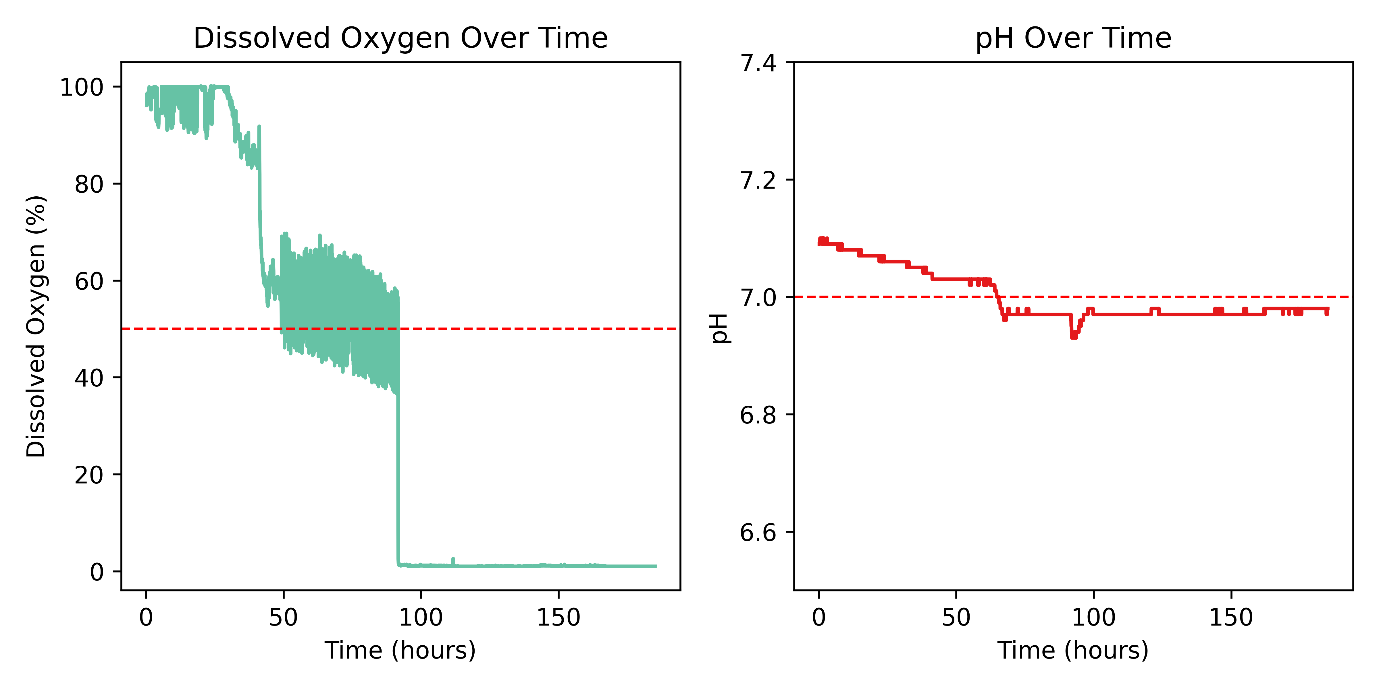


**Fig. S4. Dissolved oxygen and pH of bioreactor during cell culture.**

Dissolved oxygen was maintained at 50% (with reference to saturation with air), before gassing was stopped to induced hypoxic response.


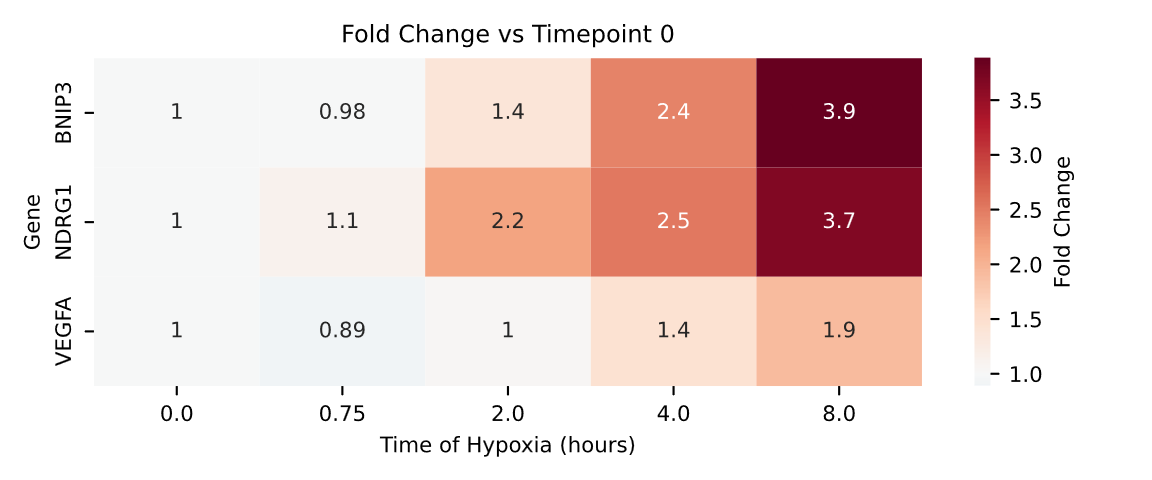


**Fig. S5.** **Temporal fold changes of scRNA-Seq mean expression values relative to the pre-hypoxic baseline (timepoint 0).**

The transcriptional upregulation of VEGFA occurs after BNIP3 and NDRG1.

**Fig. S6. Pearson correlation between pseudo-temporal cell orders of hypoxia, as calculated by a range of hypoxia marker set sizes.**


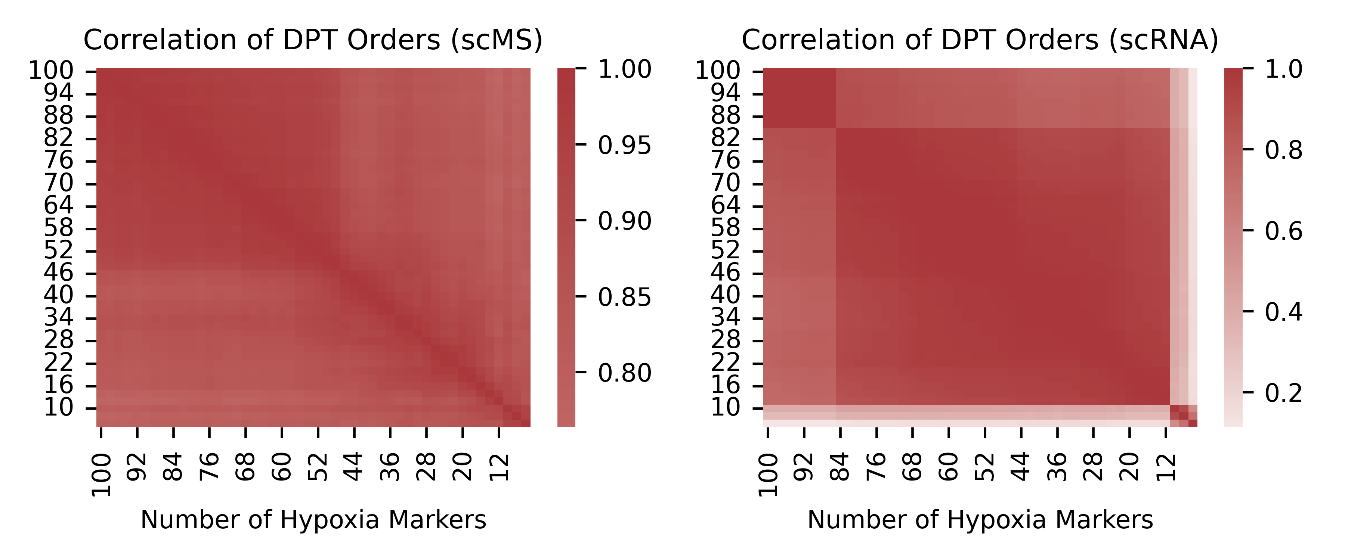

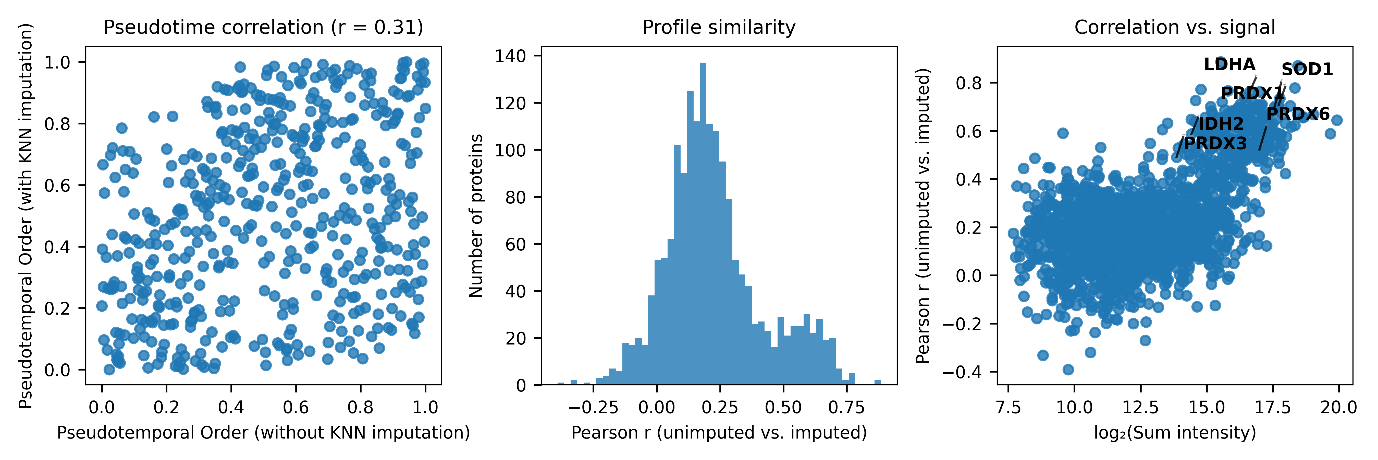


**Fig. S7. Comparing hypoxia profiles derived from raw data to kNN-smoothed data.**

The most abundant proteins retain high concordance between the two pseudo-temporal orders.
